# Carbamylated vimentin represents a relevant autoantigen in Latin American (Cuban) rheumatoid arthritis patients

**DOI:** 10.1007/s00296-016-3472-9

**Published:** 2016-04-02

**Authors:** Goitybell Martínez, Jorge A. Gómez, Holger Bang, Lorena Martínez-Gamboa, Dirk Roggenbuck, Gerd-Rüdiger Burmester, Barbara Torres, Dinorah Prada, Eugen Feist

**Affiliations:** Department for Rheumatology and Clinical Immunology, Charité University Hospital, Berlin, Germany; Immunology Laboratory, National Center of Medical Genetic, Havana, Cuba; National Center of Rheumatology, Havana, Cuba; Orgentec Diagnostika GmbH, Mainz, Germany; Faculty of Science, Brandenburg University of Technology Cottbus-Senftenberg, Senftenberg, Germany; Medipan GmbH, Dahlewitz/Berlin, Germany

**Keywords:** Rheumatoid arthritis, Anti-mutated citrullinated vimentin, Citrullinated vimentin, Carbamylated vimentin, Anti-cyclic citrullinated peptides

## Abstract

Smoking produces substances that activate proinflammatory, prothrombotic and vasoconstrictive mediators via posttranslational carbamylation of proteins. As a new consequence of carbamylation, induction of anti-carbamylated autoantibodies were observed in rheumatoid arthritis (RA) patients, sometimes prior to onset of the disease. The overall aim of this study was to characterize the reactivity of different isotypes of autoantibodies against carbamylated antigens of vimentin in relation to established rheumatoid factor (RF), anti-citrullinated protein antibodies (ACPA) and markers of disease activity in a so far largely uncharacterized population of Latin American (Cuban) patients with RA. Antigenic properties of carbamylated vimentin as well as vimentin peptides were analyzed in 101 patients with RA, 50 disease controls and 51 healthy controls. The diagnostic performance was compared with established commercial ELISA rheumatoid factor, anti-cyclic citrullinated peptide antibodies of second generation (anti-CCP2) and anti-mutated citrullinated vimentin (anti-MCV) antibodies. Prevalence of anti-MCV IgG (86 %), anti-carbamylated vimentin (carbVIM) IgG (77 %) and anti-carbamylated MCV (carbMCV) IgG antibodies (65 %) was higher than the classical RF IgM (60 %) and anti-CCP2 IgG (52 %) in this RA cohort. Of note, smoking status was associated with positive IgG antibody reactivity against CCP2 in 75.0 % and against MCV in 90 % of patients. Furthermore, IgM antibody response against carbMCV and carbVIM was observed in 80 and 90.0 % of smokers, respectively. Due to a high sensitivity of the IgM antibody isotype of anti-carbVIM of 85.2 %, the combination of ACPA with anti-carbVIM IgM provided the best diagnostic performance so far achieved in a RA cohort of this ethnic origin. We demonstrate a high prevalence of anti-carbVIM antibodies and correlation with smoking in Latin American (Cuban) RA patients. Anti-carbVIM IgM represents an useful marker in ACPA-negative patients and, in combination with ACPA IgG assays, optimizes the strategy for autoantibody testing.

**Electronic supplementary material:**

The online version of this article (doi:10.1007/s00296-016-3472-9) contains supplementary material, which is available to authorized users.

## Introduction

Posttranslational modification (PTM) of self-antigens is a key characteristic of rheumatoid arthritis (RA) [1–5]. During the last decade, especially the relevance of protein citrullination has been in the focus of intensive research. Finally, anti-citrullinated protein antibodies (ACPA) were included as a serological marker in the new American College of Rheumatology/European League against Rheumatism (ACR/EULAR) classification criteria for RA [6]. The majority of studies showed that ACPA can discriminate between RA and other arthropathies with high specificity. They may allow early identification of patients who require more aggressive therapy [7–10]. One of the most common antigenic targets employed for ACPA testing are second-generation cyclic citrullinated peptides (CCP2) with higher specificity and sensitivity than IgM rheumatoid factor (RF) test [7, 8]. However, to improve RA diagnostic, the different markers are used in combinations [11].

Nevertheless, there is still a serologic gap with seronegative patients in established RA and more emphasis was placed on the identification of novel markers. In this context, several citrullinated proteins have been identified in the synovium of inflamed joints of RA patients including fibrinogen, enolase, fibronectin, type II collagen and vimentin [9]. All of them are considered potential relevant candidates to trigger ACPA production in genetically susceptible individuals.

The first anti-citrullinated vimentin antibodies detected in RA patients were termed anti-Sa and are characterized by high specificity but low sensitivity [9, 12]. Bang et al. [13] identified an antigenic mutated isoform of vimentin, which was the basis for the introduction of the anti-mutated citrullinated vimentin assay (anti-MCV). Several studies have shown that the anti-MCV assay is a useful diagnostic tool for RA [9, 14, 15]. However, due to the overlap of ACPA specificities the diagnostic issue of seronegative patients was not solved.

Recently, carbamylation of lysine residues to form homocitrulline, another PTM, was described as a key mechanism triggering inflammatory responses by generating neoepitopes, which are targeted specifically by anti-carbamylated protein antibodies (anti-carbP) from sera samples of RA patients. The presence of anti-carP antibodies in RA patients is associated with more severe joint damage and was identified prior to the onset of the specific RA symptoms. In contrast to citrullination, carbamylation is a nonenzymatic PTM, related to aging and lifestyle such as smoking status [3–5]. Of note, smoking is responsible for high rates of preventable mortality in Cuba. Of deaths recorded in 1995 and 2007, 15 and 18 % of preventable deaths were attributed to smoking, respectively [16].

Latin America RA cohorts have been incompletely characterized so far, since in most studies only the anti-CCP2 assay has been used [17–20]. To our knowledge, there are no published studies with Cuban patients evaluating response to different isotypes of ACPA or anti-carbP antibodies. However, this population is unique due to the mixed ethnical and special environmental background. Therefore, we evaluated the reactivity of different isotypes of autoantibodies against citrullinated and carbamylated antigens derived from the sequence of vimentin and compared that with RF and anti-CCP2 in a well-characterized cohort of Cuban patients with RA. Furthermore, correlation of autoantibodies titers with disease activity and association with smoking status was investigated in this study.

## Materials and methods

### Patients

In this study, 101 patients from the National Center for Rheumatology in Cuba fulfilling the ACR classification criteria for RA [6] and 50 disease controls from the National Center of Medical Genetic were recruited: 11 undifferentiated arthritis, 10 systemic lupus erythematosus, 7 mixed connective tissue disease, 3 psoriatic arthritis, 2 spondyloarthritis, 2 autoimmune myositis, 3 systemic sclerosis, 1 polymyalgia rheumatica, 1 Sjögren’s syndrome, and 10 viral hepatitis-related arthralgia. Furthermore, 51 healthy donors served as controls (Havana Provincial Blood Donation Center). Data collection and clinical examinations were performed by a rheumatologist. Patients with RA were classified according to disease duration as early (<12 months) or established RA (>12 months). Clinical characterization of patients with RA is shown in supplementary Table S1 including disease activity score using 28 joint counts (DAS28) based on erythrocyte sedimentation rate (ESR). None of the patients with RA was treated with a biological therapy.

The study was approved by the Medical Ethics Committee of the National Center of Medical Genetic and the National Center of Rheumatology in Cuba. Written informed consent was obtained from all patients.

### Detection of RF, anti-CCP2 and anti-MCV IgG antibodies by quantitative enzyme-linked immunosorbent assay (ELISA)

Profiling of autoantibodies was performed at the research laboratory of the Department for Rheumatology and Clinical Immunology at the Charité-University Hospital in Berlin. Anti-MCV IgG antibodies and RF of different isotypes (IgM, IgG and IgA) were measured using commercially available ELISA kits (Orgentec Diagnostika, Germany), with a cutoff point at 20 U/mL. Detection of anti-CCP2 antibodies by ELISA was performed according to the manufacturer’s instructions (MEDIPAN, Germany) with the cutoff point at 30 U/mL as recommended.

### Detection of antibodies against citrullinated and carbamylated antigens of vimentin

Human recombinant vimentin, cloned, expressed and purified as described previously [13], was used to generate the citrullinated and carbamylated isoforms.

Each arginine residue from the vimentin protein was converted into a citrulline residue in vitro by rabbit muscle peptidylarginine deaminase (PAD) (Sigma; 40 U of PAD per milligram of vimentin) after incubation for 3 h at 55 °C in a buffer containing 0.1 M Tris–HCl (pH 7.6), 10 mM CaCl_2_, and 5 mM dithioerythritol. After termination of the reaction by adding EGTA (pH 8.0; final concentration of 50 mM), the samples were separeted by electrophoresis and noncitrullinated and citrullinated proteins were detected by immunoblotting with commercial anti-vimentin (H-84; Santa Cruz Biotechnology Inc., Heidelberg, Germany), and using an ACPA sera pool from the previously published cohort to verify the citrullination [21].

Carbamylated vimentin (carbVIM) and carbamylated mutated citrullinated vimentin (carbMCV) were generated after treatment of 1 ml of vimentin (1.7 mg/ml) with 500 mM potassium cyanate for 2 h at 37 °C. Excess of chemical reagents were removed from both vimentin isoforms, with desalting mini columns according to manufacturer’s instructions (zeba Spin, Thermo Scientific, Germany). Carbamylated samples were digested with trypsin, and the mass spectra of the tryptic peptides were measured with a MALDI-TOF mass spectrometer (Ultraflex III TOF/TOF, Bruker Daltonics, Proteosys AG, Mainz, Germany). The *m*/*z* values of the peptides were compared with protein databases (NCBI, Mascot browser), and as a result, the tryptic peptides were determined to be assigned proteins (peptides mass fingerprint, PMF). In addition, MS/MS sequencing were (with the Ultraflex III TOF/TOF) performed on selected peptides. Calculation of relative amounts of lysine and homocitrulline residues in each peptide was done by comparison with the quantitated synthetic peptide standard. Representative sequences and calculation of two peptides were shown: Peptide 101–113, Lys104 = 83 % and peptide 187–196, Lys189 = 82 % carbamylation.

MCV, carbVIM and carbMCV were coated on microtiter plates as earlier described by Bang et al. [21] and in accordance with the general protocol for the Orgentec immunometric enzyme immunoassay. Briefly, 1 µg/ml coating solution of modified protein in 50 mM carbonate buffer, pH = 9.0 were incubated overnight at 4 °C (100 μl/well, Costar microtiter plates). Possible over pluses of peptides/antigens were eliminated by washing the cavities with 200 µl/well of 0.1 % Tween 20 in phosphate-buffered saline and finally blocked with a 1 % bovine serum albumin solution in phosphate-buffered saline. Finally, flicking and slapping removed any residual solution.

For ELISA, 1:100 diluted serum samples (sample buffer, phosphate-buffered saline, plus 1 % bovine serum albumin and 0.05 % Tween-20) were incubated for 30 min (100 μl/well), washed three times (300 μl/well), horseradish peroxidase conjugated antihuman IgG, IgM, IgA (Dianova, Hamburg, Germany) was added and incubated for 15 min (100 μl/well). Visualization was done by incubation with 3,3′,5,5′-tetramethyl benzidine (TMB) substrate for 15 min (100 μl/well), and the reaction was terminated by adding 100 μl stop solution (0.5 mol/l H_2_SO_4_) to each well. Optical densities (OD) were measured at 450/620 nm in a BioTek micro well photometer (Synergy HT, BioTek Instruments Inc, USA) and transformed to units per milliliter using a 4-parameter-fit titration curve.

All steps were carried out at room temperature. Each serum sample was tested in duplicate. The distribution of OD of antibodies against modified peptides in healthy individuals (*n* = 112) was assessed, and values >98th percentile of the healthy controls were considered positive for all antibodies.

Antibodies against modified vimentin peptides were determined according to the recently published procedure [21]. Briefly, a vimentin peptide sequence (used peptide sequence is bold and underlined in the shown N-terminal vimentin sequence) known to be preferential recognized by autoantibodies from RA patients was used for designing a panel of modified peptides [22, 23].
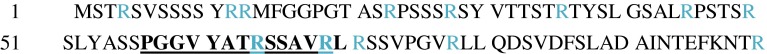


The peptide sequence: NH2-GGVYAT**X**SSAVR-OH was used as “native vimentin” reference peptide P62. The glycine residue in position 2 was mutated into an arginine amino acid residue to increase the sensitivity of the citrullinated peptide. The designated position X was substituted either with the citrulline (citrullinated) or with the homocitrulline (carbamylated) amino acid residue. The N-terminal biotinylated peptides, identical in length (12 amino acids) and composition, but differing only in position 7 were synthesized (JPT Peptide Technologies GmbH, Berlin, Germany; details are illustrated in supplementary figure S2).

Coating and assay performance were done as described above, with the following amendments: Only 0.5 µg/ml biotinylated peptide was mixed in phosphate-buffered and coated. Possible over pluses of peptides was eliminated by washing the cavities with 200 µl/well of 0.05 % Tween 20 in phosphate-buffered saline. Finally, flicking and slapping removed any residual solution.

### Statistical analysis

Statistical analysis was performed using GraphPad Prism software version 6.0 and Epidat 3.1. Data were expressed as median and interquartile ranges between the 25th quartile and 75th quartile for continuous variables and as frequency and percentage for categorical variables. The nonparametric Mann–Whitney* U* test was applied when two groups were compared. Sensitivity, specificity, predictive values, Youden index (YI) and areas under curve (AUC) of Receiver Operating Characteristic Curve (ROC) analysis were determined. The correlation analysis was performed according to Spearman’s test. For association analysis *χ*^2^ and odd ratios (OR) were calculated. *p* values <0.05 were considered significant.

## Results

### Generation of recombinant citrullinated and/or carbamylated vimentin isoforms

Tobacco smoke contains pathogenic ingredients, i.e., hydrocyanic acid (OCN-), which is normally rapidly detoxified by transsulfuration forming thiocyanate ions (-SCN). These thiocyanate ions can be oxidized in vivo and subsequently form cyanide in inflammatory loci through the action of hydrogen peroxide plus myeloperoxidase (MPO) from neutrophils or eosinophil peroxidase (EPO). Therefore, we established the procedure for generation of carbamylated vimentin with the nonenzymatic incubation of potassium cyanate (KCN). As shown in supplementary figure S5, nearly quantitative carbamylation (approximately 80 %) of vimentin could be verified by amino acid analysis using MALDI-TOF mass. Furthermore, no additional modifications of other amino acid residues were detectable indicating that only homocitrulline residues were formed from lysine residues after KCN- exposure.

The procedure of vimentin citrullination was established with a very low amount of PAD as described previously [13]. Cross-reaction with the PAD enzyme was excluded as shown by Western blot analysis in supplementary figure S3. To generate a double-modified vimentin isoform, a sequential procedure was established by citrullinating the mutated vimentin in a first step, followed by carbamylation.

The efficiency of each step was verified by SDS-PAGE electrophoresis with coomassie brilliant staining showing a shift to higher molecular weight for the citrullinated isoform and a backward shift after additional carbamylation (supplementary figure S4). To confirm that the migration shift was due to citrullination, blots containing the same samples were analyzed using affinity purified anti-citrulline antibody from our previously study (supplementary figure S2 B) [21]. The results showed that the citrullinated and the citrullinated plus carbamylated isoform cross-react with the purified antibodies. These results are in agreement with the distinct modification positions for citrullination (modified arginine R) and carbamylation (modified lysine K), as illustrated in supplementary figure S6.

### Autoantibodies reactivity against RF and anti-CCP2

**Fig. 1:**
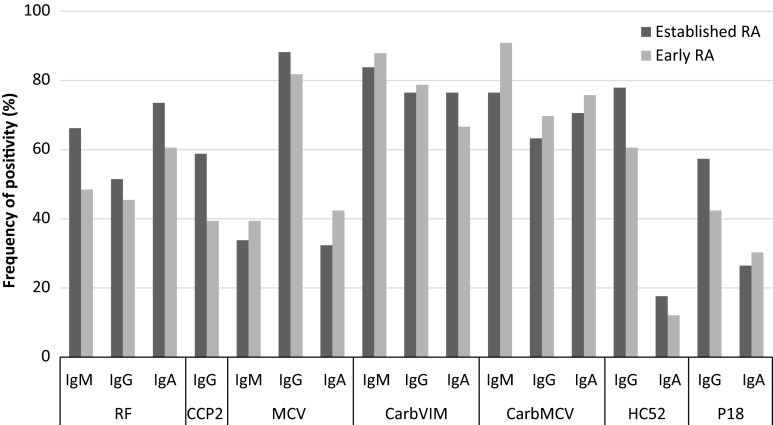
Relative frequency of antibodies against citrullinated and carbamylated sequences of vimentin, anti-CCP2 and RF in patients with early and established RA. *RA* rheumatoid arthritis, *RF* rheumatoid factor, *CCP2* cyclic citrullinated peptides of second generation, *MCV* mutated citrullinated vimentin, *carbVIM* carbamylated vimentin, *carbMCV* carbamylated mutated citrullinated vimentin, *CCP VIM* cyclic citrullinated peptides of vimentin, *Ig* immunoglobulin. Cutoff value of anti-CCP2 was 30 U/mL according to manufacturer’s instruction and for others assay was 20 U/mL

**Fig. 2:**
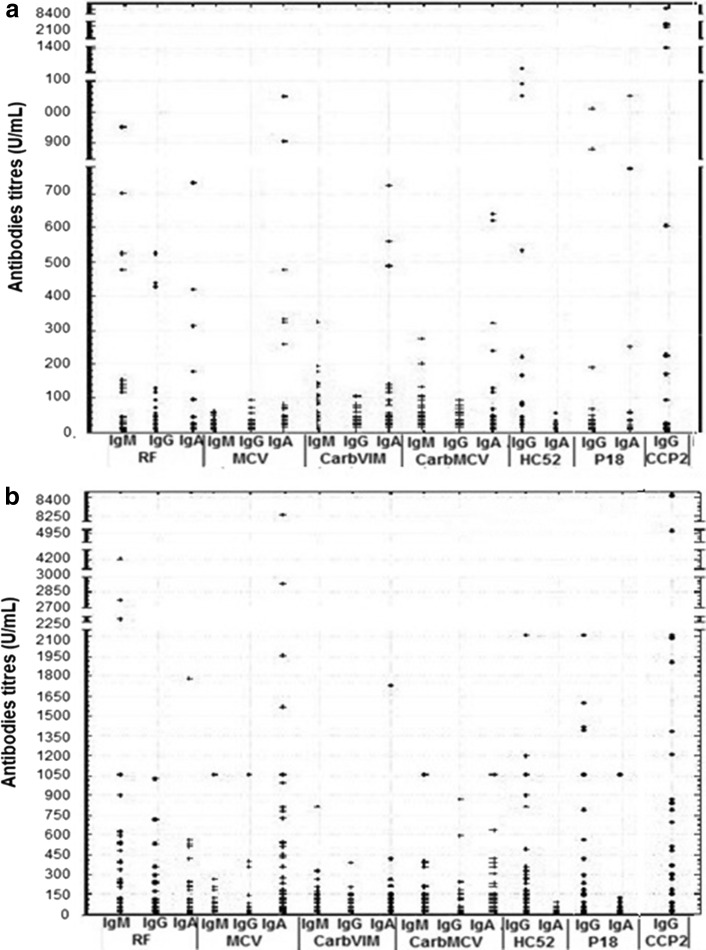
Median of titers of antibodies against citrullinated and carbamylated sequences of vimentin, anti-CCP2 and RF in RA patients. **a** Early RA. **b** Established RA. RA: rheumatoid arthritis. *RF* rheumatoid factor, *CCP2* cyclic citrullinated peptides of second generation, *MCV* mutated citrullinated vimentin, *carbVIM* carbamylated vimentin, *carbMCV* carbamylated mutated citrullinated vimentin, *CCP VIM* cyclic citrullinated peptides of vimentin. Ig: immunoglobulin. Cutoff value of anti-CCP2 was 30 U/mL according to manufacturer’s instruction, and for others assay was 20 U/mL

By using regular assays for the detection of RF and anti-CCP2, frequencies of RF IgM (48.5 %), RF IgA (60.6 %) and RF IgG (45.5 %) were higher than anti-CCP2 (39.4 %) antibodies in patients with early RA. In patients with established RA, prevalence of RF IgM (66.0 %) and RF IgA (73.5 %) was also higher than anti-CCP2 (58.8 %) followed by RF IgG (51.5 %). The most frequently detected standard autoantibody in this cohort was RF IgA (Fig. 1). Of note, higher autoantibody titers were observed in patients with established RA (Fig. 2b) compared to early disease (Fig. 2a).

### Autoantibodies reactivity against carbamylated and citrullinated antigens based on the sequence of vimentin

The reactivity against carbamylated and citrullinated vimentin antigens showed a higher prevalence compared to anti-CCP2 and RF in Cuban patients with RA (Fig. 1). Interestingly, patients with early RA showed highest frequencies for anti-carbMCV IgM (90.9 %) and anti-carbVIM IgM (87.9 %) followed by anti-MCV IgG (81.8 %). In patients with established RA, these antibodies were also predominant with the highest prevalence for anti-MCV IgG (88.2 %) followed by anti-carbVIM IgM (83.8 %) and anti-carb MCV IgM (76.5 %).

Patients with RA showed also higher frequencies of IgG autoantibodies against the carbamylated peptide of vimentin HC52, compared to RF IgM, RF IgG and anti-CCP2 (Fig. 1). However, these frequencies were lower than those observed for anti-carbVIM IgM. Thus, reactivity against citrullinated and carbamylated isoforms of vimentin was higher than those observed against modified peptides. Overall, reactivity against newly found citrullinated and carbamylated sequences of vimentin was more frequent with IgM and IgG antibodies than with IgA.

### Demographic, clinical characteristics and autoantibody reactivity of RA patients, depending on the positivity for anti-MCV IgG

Patients with anti-MCV IgG antibodies showed no statistical difference in demographic characteristics compared to the seronegative cohort (Table 1). Nevertheless, clinical phenotypes were different in these two groups of patients with a higher rate of low DAS28 and remission achieved in anti-MCV negative patients (*p* = 0.0048, OR 0.2, 95 % CI 0.6–0.1)

**Table 1:** Demographic and clinical characteristics of RA patients, according to presence of anti-MCV IgG antibodies

Assay	Early RA (*n* = 33)		Established RA (*n* = 68)	
	MCV+ (*n* = 27)	MCV− (*n* = 6)	MCV+ (*n* = 60)	MCV− (*n* = 8)
F, *n* (%)	19 (70.4)	5 (83.3)	52 (86.7)	6 (75.0)
Age in years,* m* (IQR)	50 (41–59)	43 (32–48)	52 (43–51)	57 (48–71)
Smoker, *n* (%)	7 (25.9)	1 (16.7)	11 (18.3)	1 (12.5)
Disease duration in years,* m* (IQR)	1.0 (0.5–1.0)	0.9 (0.8–1.0)	6.0 (3.0–11.0)	11.0 (5.0–16.2)
DAS28,* m* (IQR)	5.3 (4.2–6.3)	3.3 (3.0–4.9)	5.1 (3.8–5.9)	5.0 (4.5–5.7)
Remission: DAS28 ≤ 2.6; *n* (%)	1 (3.7)	0	6 (10.0)	0
Low: DAS28 > 2.6 and ≤ 3.2, *n* (%)	2 (7.4)	3 (50.0)	7 (11.7)	5 (62.5)
Moderate: DAS28 > 3.2 and ≤ 5.1, *n*(%)	9 (33.3)	1 (16.7)	19 (31.7)	5 (62.5)
High: DAS28 > 5.1, *n* (%)	15 (55.6)	2 (33.4)	28 (46.7)	3 (37.5)
HAQ,* m* (IQR)	0.60 (0.44–0.81)	0.28 (0.25–0.24)	0.75 (0.25–1.37)	1.06 (0.81–1.41)
ESR,* m* (IQR)	44.0 (25.5–64.5)	16.0 (13.5–21.5)	29.0 (16.0–54.2)	26.0 (16.3–62.8)
CRP positivity, *n* (%)	21 (77.8)	1 (16.7)	36 (60.0)	2 (25.0)

*RA* rheumatoid arthritis, *n* number of patients, *DAS28* disease activity score, *ESR* erythrocyte sedimentation rate, *CRP* C-reactive protein, *HAQ* Health Assessment Questionnaire Index, *m* median, *n* number of patients, *IQR* interquartile range

* Significant differences between anti-MCV IgG positive and negative groups on median (*p* < 0.05)

By comparing the titers of the RF IgM, anti-CCP2 and anti-carbVIM IgM, we observed higher values for anti-MCV IgG positive compared to negative patients (Table 2). Differences were only significant for RF IgM and anti-CCP2 antibodies in both early (*p* < 0.01) and established (*p* < 0.05) RA patients. A high coincidence rate of autoantibodies was especially observed for combinations of anti-carbVIM IgM and anti-MCV IgG in patients with early (up to 72.7 %), and established RA (up to 75.0 %). Out of 25 patients tested negative for all isotypes of RF and anti-CCP2, 18 were positive for anti-MCV IgG (Table 2).

**Table 2:** Comparison of level and frequencies of positivity of autoantibodies according to presence of anti-MCV IgG

Assay	Early RA (*n* = 33)		Established RA (*n* = 68)	
	MCV+ (*n* = 27)	MCV− (*n* = 6)	MCV+ (*n* = 60)	MCV− (*n* = 8)
RF IgM+, *n *(%)	16 (48.5)	0	43 (63.2)	0
RF IgM,* m* (IQR)*	27.2 (10.5–303.2)	6.9 (2.3–12.3)	66.8 (15.3–316.7)	4.35 (0–21.9)
CCP2 +, *n* (%)	13 (39.3)	0	40 (58.8)	0
CCP2,* m* (IQR) *	22.6 (4.1–2100.0)	4.5 (3.2–7.1)	163.7 (79–1772.0)	3.8 (1.9–5.6)
CarbVIMIgM+, *n* (%)	24 (72.7)	5 (83.3)	51 (75.0)	6 (75.0)
CarbVIMIgM,* m* (IQR)	63.6 (10.1–126.8)	43.9 (25.2–97.7)	55.1 (30.6–109.5)	31.0 (20.7–65.6)
RF IgM−/CCP2−, *n* (%)	10 (30.3)	6 (100)	15 (22.1)	6 (75.0)
RF IgM +/CCP2 +, *n* (%)	12 (36.4)	0	38 (55.9)	0
RF IgM, IgG, IgA−/CCP2−, *n* (%)	6 (18.2)	5 (83.3)	12	2 (25.0)
RF IgM, IgG, IgA +/CCP2 +, *n* (%)	10 (30.3)	0	27 (45.0)	0
RF IgM, IgG, IgA +/CCP2−, *n* (%)	2 (6.1)	0	4 (5.9)	0
CCP2−/CarbVIMIgG +/HC52 IgG +, *n* (%)	1 (3.1)	1 (16.7)	10 (7, 16)	2 (25.0)
CarbVIMIgM +/carbMCVIgM+, *n* (%)	24 (72.7)	5 (83.3)	47 (69.1)	5 (62.5)
CarbVIMIgM −/carbMCVIgM−, *n* (%)	2 (6.1)	0	9 (13.2)	2 (25.0)
CarbVIMIgM +/carbMCVIgM +/RF IgM +/CCP2 +, *n* (%)	12 (36.4)	0	30 (44.1)	0

*RA* rheumatoid arthritis, *MCV* mutated citrullinated vimentin, *CarbVIM* carbamylated vimentin, *CarbMCV* carbamylated mutated citrullinated vimentin, *RF* rheumatoid factor, *CCP2* cyclic citrullinated peptides of second generation, *Ig* immunoglobulin, *n* number of patients, *m* median, *IQR* interquartile ranges

* Significant differences between anti-MCV IgG positive and negative groups on antibodies titers (*p* < 0.05)

From the patients with negative results for anti-CCP2, anti-carbamylated antigens IgG antibodies were detectable in 13.9 and 3.0 % of cases against the protein and peptide, respectively. Concurrent seropositivity for anti-carbVIM IgM, anti-carbMCV IgM, anti-MCV IgG, RF IgM and anti-CCP2 was detectable in 12.0 % of samples from patients with early RA and in 30.0 % of patients with established RA (Table 2). Of note, all anti-MCV seronegative patients were also negative for RF IgM and anti-CCP2 assays in both early and established RA.

**Fig. 3:**
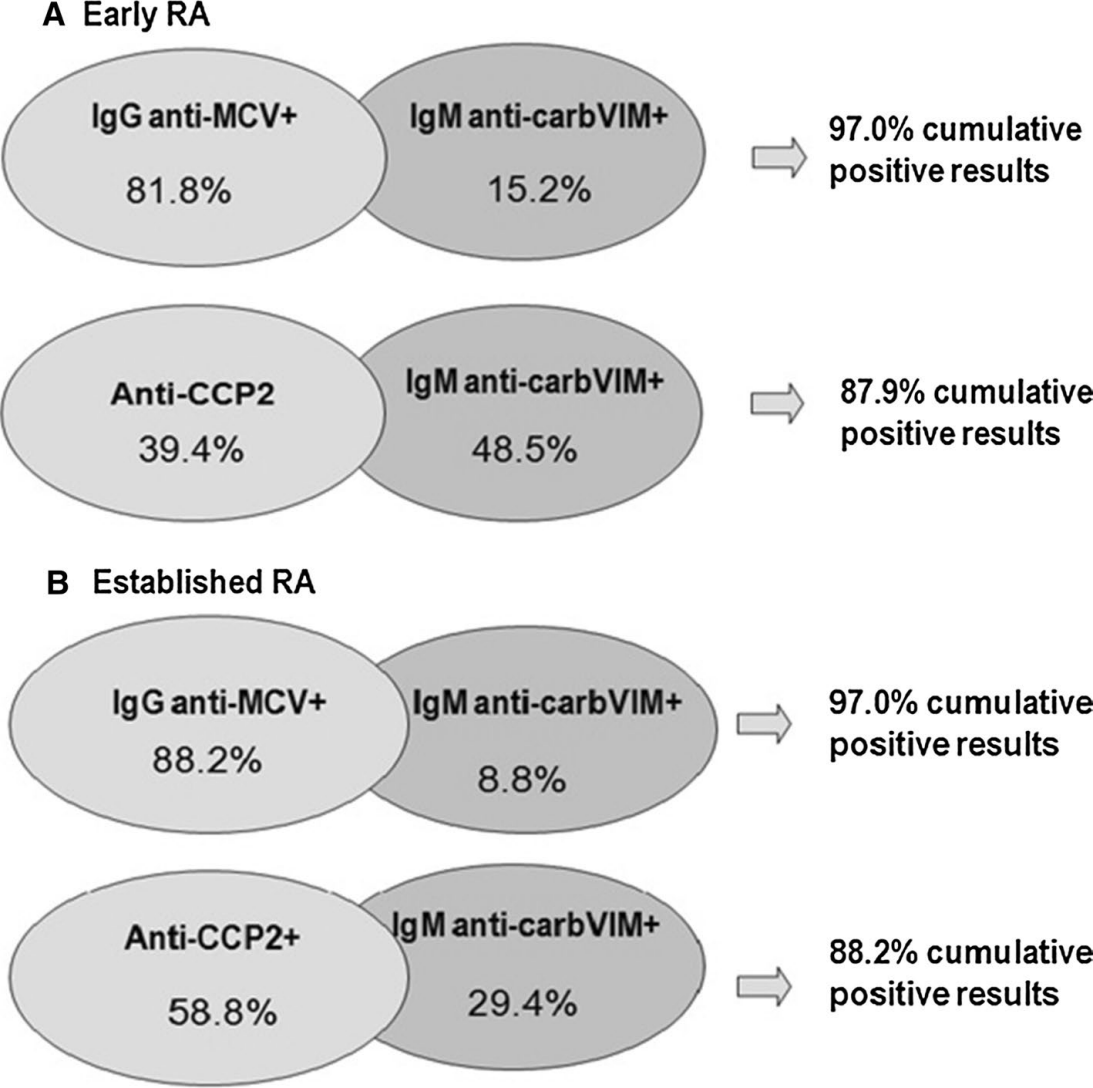
Cumulative positive results of ACPA and anti-carbVIMIgM assays in patients with early and established RA. *RA* rheumatoid arthritis, *MCV* mutated citrullinated vimentin, *carbVIM* carbamylated vimentin, *Ig* immunoglobulin. Positive results were considered according to the manufacturer’s instructions

In anti-MCV IgM-negative patients, determination of anti-carbVIM IgM identified an additional proportion of 15.2 % of positive early RA cases and 8.8 % of patients with established disease. Combination of these two assays was able to detect autoantibodies in 97 % of patients with early or established RA (Fig. 3). In contrast, combination of anti-CCP2 and anti-carbVIM IgM assay was able to detect autoantibodies at a lower frequency.

### Diagnostic value of anti-MCV IgG, anti-carbVIM IgM and anti-CCP2 assays

The diagnostic sensitivity and specificity of each assay using different cutoffs and ROC analysis are shown in Table 3. Optimal cutoff value, based on the highest possible sensitivity and specificity, using YI, was the same as recommended by the manufacturer for anti-MCV assay IgG; for anti-carbVIM IgM cutoffs of 20 and 30 U/mL showed very similar balance of sensitivity and specificity and for anti-CCP2 optimal cutoff was 40 U/mL.

**Table 3:** Diagnostic value of ACPA and anti-carbVIMIgM assays using Receiver Operating Characteristic Curve analysis

Assay, area under curve (confidence interval)	Cutoff (U/mL)	Se	Sp	PPV	NPV	YI
Anti-carbVIMIgM, 0.9111 (0.8722–0.9501)	20	85.2	90.1	89.6	85.9	0.75
	30	78.2	96.3	96.3	81.8	0.75
	40	59.4	100	100	71.7	0.59
Anti-MCV IgG, 0.8479 (0.7959–0.8999)	20	86.1	75.3	77.7	84.4	0.61
	30	68.3	87.1	84.1	73.3	0.55
	40	60.4	92.1	88.4	69.9	0.52
Anti-CCP2, 0.7537 (0.6953–0.8122)	20	54.5	92.1	87.3	66.9	0.47
	30	52.5	95.1	91.4	66.9	0.48
	40	51.5	98.1	96.3	66.9	0.50
Anti-carbVIMIgM and anti-MCV IgG	20	74.2	96.0	94.9	78.9	0.70
Anti-carbVIMIgM or anti-MCV IgG	20	97.0	69.3	76.0	95.9	0.66
Anti-carbVIMIgM and anti-CCP2	20/40	46.5	99.0	97.9	64.9	0.46
Anti-carbVIMIgM or Anti-CCP2	20/40	91.1	89.1	89.3	90.9	0.80

*MCV* mutated citrullinated vimentin, *carbVIM* carbamylated vimentin, *Ig* immunoglobulin, *Se* sensitivity, *Sp* specificity, *PPV* positive predictive value, *NPV* negative predictive value, *YI* Youden index

A higher sensitivity was observed by using combination of assays, when positive results were considered for at least one assay anti-carbVIM IgM or anti-MCV IgG (97.0 %). For combination of anti-carbVIM IgM or anti-CCP2, the sensitivity was 91.1 %. Interestingly, specificity observed for combination of anti-carbVIM IgM and anti-CCP2, when positive results were considered for both assays, was higher (99.1 %) than specificity observed for combination of anti-carbVIM IgM and anti-MCV IgG (96.0 %). The highest possible sensitivity and specificity balance using YI was observed for combination of assays, when positive results were considered for at least one of both: anti-carbVIM IgM and anti-CCP2 assays. Of note, the AUC of ROC analysis was superior for anti-carbVIM IgM assay. The reactivity of anti-MCV IgG, anti-carbVIM IgM and anti-CCP2 assays in disease and healthy controls are shown in supplementary table S7.

In patients with established RA, a weak correlation with DAS28 was only observed for anti-CCP2 assay (supplementary table S8). Low DAS28 and remission were more frequently achieved in anti-CCP2-negative patients (*p* = 0.0091, OR 0.2, 95 % CI 0.7–0.1).

Interestingly, 75.0 % of patients positive for anti-CCP2, 90 % of patients positive for anti-MCV, 80 % of patients positive for anti-carbMCV IgM and 90.0 % of patients positive for anti-carbVIM IgM were smokers. A significant association was only evident for smoking status and presence of anti-CCP2 (*p* = 0.0216, OR 3.9, 95 % CI 1.3–11.9), for CRP and RF IgM (*p* = 0.0001, OR = 6.4, 95 % CI 2.5–16.8) as well as anti-MCV IgG (*p* = 0.0307, OR = 5.3, 95 % CI 1.3–21.3) and anti-CCP2 (*p* = 0.0000, OR = 5.8, 95 % CI 2.4–14.1).

The Spearman rank correlation test was significant (*p* < 0.05) for anti-CCP2, anti-MCV IgG as well as anti-carbVIM IgM antibodies and RFIgM in both early and established RA patients. Anti-CCP2, anti-MCV IgG and anti-carbVIM IgM showed positive correlation with ESR in early RA patients (supplementary table S8).

## Discussion

The most interesting finding in our study was the high frequency of antibodies against citrullinated and carbamylated antigens of vimentin, as well as the good diagnostic performance of the anti-carbVIM IgM assay in Cuban patients with RA. This observation is of importance not only for improved diagnostic procedures, but also for a better understanding of the pathogenesis based on the different ethnic and genetic background. Adequate identification of seropositive patients and potential measurement of all three isotypes could improve the routine diagnostic procedures in RA, to close the gap of seronegative patients and to identify those with severe outcome [11]. It has been stated that patients with all isotypes of RF have a very high likelihood of been diagnosed with RA, similar to patients with simultaneous presence of RF IgM, RF IgA and anti-CCP2 antibodies [11]. In agreement with this observation, very few patients were negative for all RF isotopes and ACPA in our study.

According to our results, the frequency of RF IgM in the Cuban patients was similar to another study that used ELISA, since seropositivity of RF has been reported to be close to 50 % in Latin America [17]. Although Kokuina et al. described the presence of RF in the Cuban population at higher frequencies (84 % in established and 67 % in early RA) by using immunoturbidimetry [24], it has to be considered that these assay techniques are not directly comparable to ELISA with respect to obtained isotypes, titers and specificity [19, 20]. Interestingly, our frequencies for RF IgA were higher than RF IgM in Cuban patients. While other authors mentioned prevalence rates of RF IgA close to 42.5 % in early and 37.5 % in established RA in another country of Latin America [15], we observed 60.6 % in early and 73.5 % in established disease. This finding is of special interest since RF IgA has been associated with severity of the disease and was reported to predict the development of RA [11]. The observed higher frequencies of RF IgA can be partially explained by the fact that almost 50 % of Cuban patients in our cohort had high disease activity (DAS28 > 5.1) and about 80 % of patients had moderate or high disease activity (DAS28 > 3.2).

Although the pathogenesis of RA has not yet been elucidated, growing evidence supports the involvement of an immune response against modified antigens. Since citrullinated peptides can bind with a higher affinity to human leukocyte antigen (HLA) DR alleles encoding the so-called shared epitope, they are efficiently presented to the immune system and can lead to activation of CD4+ T cell breaking immunological tolerance to self-antigens [2].

Of note, the prevalence of anti-CCP2 antibodies was not as high as expected for early RA in our study. However, prevalence of these antibodies has been reported between 48 and 85 % in other cohorts in Latin America [17–20]. These differences can be due to genetic factors, environmental background or the commercial assay used [2, 7, 8]. Genetic alterations that are likely to be influenced by environmental factors can lead to the production of ACPA [2]. Unfortunately, there are no data available on the frequency of the shared epitope in the Cuban population.

As an ubiquitous expressed auto-antigen, vimentin might be of special relevance in triggering ACPA production. Vimentin immunization can induce an arthritogenic process as well as a specific T cell response in mice. In this context, an association of an immune response against vimentin and certain human leukocyte antigen (HLA)-DR alleles encoding the “shared epitope” has been reported. Moreover, the presence of citrullinated vimentin in immuno-complexes of synovial fluid of RA patients with ACPA and the high specificity of anti-citrullinated vimentin antibodies further emphasize their role in the pathogenesis of RA [9].

Previous studies in Mexico have reported a prevalence of anti-MCV antibodies in 81.0 % [14] and 69.7 % of cases [25]. However, both studies did not differentiate between patients with early or established diseases. In our study, we observed high frequencies of this autoantibody in both subgroups. With respect to the study by Diaz-Toscano et al. [14] showing sensitivity of 81 % and specificity of 94 % for anti-MCV assay, we observed a higher sensitivity but lower specificity in our cohort.

The B cell ACPA response is dependent on T cell activation after the first antigen contact for production of the IgM isotype and followed by the production of IgG after repeated antigen exposure. Usually, the half-life of IgM is short, and memory B cells against T-cell-dependent protein antigens typically produce IgG and not IgM antibodies. One explanation for the persistent reactivity of IgM and IgG ACPA is that some citrullinated antigens are able to activate new B cells despite concurrent recognition by IgG ACPA. In this context, it has been observed that several defined citrullinated antigens are recognized only by IgG ACPA, whereas others are also recognized by IgM ACPA [26]. In our cohort, the prevalence of anti-MCV IgG was higher than anti-MCV IgM and anti-MCV IgA supporting the usefulness of IgG ACPA for diagnostic purposes.

Initial evidence suggests that carbamylation of antigens is also involved in the pathogenesis of RA. Carbamylated proteins induce the chemotaxis and activation of CD4+ T cells with a strong antibody response. This could be the first phase of autoreactivity in RA subsequently leading to the local recognition of citrullinated peptides within the joints and to the development of erosive arthritis [27, 28]. Shi et al. [3, 4] identified anti-carbP antibodies in the serum of RA patients that predict disease progression in ACPA-negative patients. Also Stoop et al. [27] observed an immune response against carbamylated antigens in an arthritis animal model induced by collagen before disease onset. In agreement with Shi et al. [3], we also found anti-carbVIM and anti-carbMCV antibodies in both ACPA-positive and ACPA-negative patients with RA. In subsequent studies, it would be of interest to clarify, whether antibodies against carbamylated antigens can also predict the development of RA independently of the presence of ACPA [4].

According to our observations, the anti-carbVIM assay was able to detect more anti-CCP2 seronegative patients in early than in established RA. These observations are in agreement with the data obtained by Stoop et al. [27], indicating that the tolerance to carbamylated proteins, in contrast to the response to citrullinated proteins, is easily broken and that arthritis boosts the immune response against these proteins explaining the phenomenon that anti-carbP antibodies already appeared before disease onset [27].

There is growing evidence that diagnostic performance in RA patients could be increased by using more than one assay [8, 29]. In fact, we observed that the sensitivity could be increased by using both anti-MCV IgG and anti-carbMCV IgM or anti-CCP2 and anti-carbMCV IgM assays. Such a very high sensitivity by combination of tests is extremely relevant and could significantly facilitate early diagnosis of RA. Although reactivity against modified vimentin was predominant, the modified peptides were also recognized as autoantigens by antibodies from patients with early and established RA.

Several studies have shown a stronger correlation of anti-MCV levels with clinical parameters compared with anti-CCP2 levels [2, 30]. Other authors suggest that anti-carbP antibodies can have clinical value in the prediction of RA, as well as for disease progression [3, 27, 31]. We found similar correlations between anti-MCV IgG, anti-CCP2 and anti-carbVIM IgM antibodies with ESR in patients with early RA. It is of note that we observed a weak correlation between anti-CCP2 antibodies and DAS28 in patients with established disease in contrast to other studies [32, 33]. Furthermore, seronegative RA patients for anti-CCP2 or anti-MCV were more frequently in low disease activity and remission. Thus, we observed two clinical phenotypes according to the presence or absence of anti-MCV or anti-CCP2 antibodies.

Pathogenic models of RA nowadays include smoking as a relevant trigger factor of immune reactions to autoantigens modified by citrullination in patients with HLA-DR shared epitope [2]. This is in agreement with the observed significant association between anti-CCP2 antibodies and smoking status as well as disease activity in our study. Although causative association are not ideal in a cross-sectional design, we were not able to show an association between smoking and the formation of antibodies against carbamylated antigen. While exposure to smokes causes carbamylation at physiological levels depending on the concentrations, there are potentially also other so far not identified triggering factors for the induction of carbamylation in RA [31, 34].

Taken together, combination of two tests for ACPA and anti-carbVIM IgM significantly increases the diagnostic sensitivity for RA and provides the best performance achieved so far in a Cuban cohort. Thus, for anti-MCV IgG or anti-CCP2 seronegative patients it can be recommended to test for anti-carbVIM IgM. This strategy should be implemented in the diagnostic of RA in Latin American countries and needs to be confirmed in other cohorts. Our results support the model that citrullination and carbamylation may contribute unidirectional to the pathogenesis of RA.

## Electronic supplementary material

Below is the link to the electronic supplementary material.

Supplementary material 1 (DOCX 505 kb)

## References

[1] Abbas AK, Lichtman A, Pillai S (2012). Cellular and molecular immunology.

[2] Wagner C, Sokolove J, Lahey L, Bengtsson C, Saevarsdottir S, Alfredsson L (2015). Identification of anticitrullinated protein antibody reactivity in a subset of anti-CCP-negative rheumatoid arthritis: association with cigarette smoking and HLA-DRB1 ‘shared epitope’ alleles. Ann Rheum Dis.

[3] Shi J, Knevel R, Suwannalai P, Van Der Linden MP, Janssen GMC, Van Veelen PA (2011). Autoantibodies recognizing carbamylated proteins are present in sera of patients with rheumatoid arthritis and predict joint damage. Proc Natl Acad Sci USA.

[4] Shi J, Van De Stadt L, Levarht N, Huizinga T, Toes R, Trouw L (2013). Anti-carbamylated protein antibodies are present in arthralgia patients and predict the development of rheumatoid arthritis. Arthritis Rheum.

[5] Shi J, Van Veelen PA, Mahler M, Janssen GM, Drijfhout JW, Huizinga TW (2014). Carbamylation and antibodies against carbamylated proteins in autoimmunity and other pathologies. Autoimmun Rev.

[6] Aletaha D, Neogi T, Silman AJ, Funovits J, Felson DT, Bingham CO (2010). Rheumatoid arthritis classification criteria: an American College of Rheumatology/European League Against Rheumatism collaborative initiative. Arthritis Rheum.

[7] Pruijn G, Wiik A, Venrooij JV (2010). The use of citrullinated peptides and proteins for the diagnosis of rheumatoid arthritis. Arthritis Res Ther.

[8] Demoruelle KM, Parish M, Derber L, Kolfenbach J, Hughes-Austin J, Weisman M (2013). Anti-cyclic citrullinated peptide assays differ in subjects at elevated risk for rheumatoid arthritis and subjects with established disease. Arthritis Rheum.

[9] Van Steendam K, Tilleman K, Deforce D (2011). The relevance of citrullinated vimentin in the production of antibodies against citrullinated proteins and the pathogenesis of rheumatoid arthritis. Rheumatology (Oxford).

[10] Demoruelle KM, Deane K (2011). Antibodies to citrullinated protein antigens (ACPAs): clinical and pathophysiologic significance. Curr Rheumatol Rep.

[11] Jaskowski T, Hill HR, Russo KL, Lakos G, Szekanecz Z, Teodorescu M (2010). Relationship between rheumatoid factor isotypes and IgG anti-cyclic citrullinated peptide antibodies. J Rheumatol.

[12] Després N, Boire G, Lopez-Longo FJ, Ménard HA (1994). The Sa system: a novel antigen-antibody system specific for rheumatoid arthritis. J Rheumatol.

[13] Bang H, Egerer K, Gauliard A (2007). Mutation and citrullination modifies vimentin to a novel autoantigen for rheumatoid arthritis. Arthritis Rheum.

[14] Díaz-Toscano ML, Olivas-Flores EM, Zavaleta-Uñiz SA, Gamez-Nava JI, Cardona-Muñoz EG, Ponce-Guarneros M (2014). Comparison of two assays to determine anti-citrullinated peptide antibodies in rheumatoid arthritis in relation to other chronic inflammatory rheumatic diseases: assaying anti-modified citrullinated vimentin antibodies adds value to second-generation anti-citrullinated cyclic peptides testing. Biomed Res Int.

[15] Zahran W, Mahmoud M, Shalaby K, Abbas M (2013). Unique correlation between mutated citrullinated vimentin IgG autoantibodies and markers of systemic inflammation in rheumatoid arthritis patients. Ind J Clin Biochem.

[16] Varona P, Herrera D, García RG, Bonet M, Romero T, Venero SJ (2009). Smoking-attributable mortality in Cuba. MEDICC Rev.

[17] Da Mota LMH, Neto LL, Pereira IA, Burlingamer R, Menard H, Laurindo IM (2011) Autoantibodies in early rheumatoid arthritis—Brasília cohort—results of a three-year serial analysis. Rev Bras Reumatol 51:558–571. www.scielo.br/pdf/rbr/v51n6/en_v51n6a04.pdf 22124591

[18] Haye MJ, Retamozo S, Vetorazzi L, Peano N, Díaz PE, Castaños MS et al (2013) Anticuerpo anticitrulina y manifestaciones extra articulares en artritis reumatoidea. Medicina (Buenos Aires) 73: 21–25. http://www.scielo.org.ar/scielo.php?pid=S0025-76802013000100004&script=sci_arttext 23335701

[19] Marcos J, Waimann C, Para FD, Hogrefe J, Retamozo S, Caeiro F (2011). General characteristics of an early arthritis cohort in Argentina. Rheumatology.

[20] Goeldner I, Skare TL, Reason I, Nisihara RM, Silva M, Shirley R (2010). Concise report Anti-cyclic citrullinated peptide antibodies and rheumatoid factor in rheumatoid arthritis patients and relatives from Brazil. Rheumatology.

[21] Juarez M, Bang H, Hammar F, Reimer U, Dyke B, Sahbudin I (2015). Identification of novel anti-acetylated vimentin antibodies in patients with early inflammatory arthritis. Ann Rheum Dis.

[22] Vossenaar ER, Despres N, Lapointe E, Van Der Heijden A, Lora M, Senshu T (2004). Rheumatoid arthritis specific anti-Sa antibodies target citrullinated vimentin. Arthritis Res Ther.

[23] Kokkonen H, Brink M, Hansson M, Lassen E, Mathsson-Alm L, Holmdahl R (2015). Associations of antibodies against citrullinated peptides with human leukocyte antigen-shared epitope and smoking prior to the development of rheumatoid arthritis. Arthritis Res Ther.

[24] Kokuina H, Chico A, Carballar L, Gutiérrez A, Soto J, Estévez M et al (2008) Rheumatoid factor: association with the radiological erosion and with the rheumatoid arthritis activity. Rev Cubana Med. www.scielo.sld.cu/scielo.php?script=sci_arttext&pid=S0034-5232008000300004&lng=es&nrm=iso&tlng=es

[25] Gonzalez-Lopez L, Rocha-Muñoz AD, Ponce-Guarneros M, Flores-Chavez A, Salazar-Paramo M, Nava A (2014). Anti-cyclic citrullinated peptide (anti-CCP) and anti-mutated citrullinated vimentin (anti-MCV) relation with extra-articular manifestations in rheumatoid arthritis. J Immunol Res.

[26] Suwannalai P, Willemze A, Van Toorn L, Stoeken-Rijsbergen G, Levarht N, Drijfhout JW (2011). The fine specificity of IgM anti-citrullinated protein antibodies (ACPA) is different from that of IgG ACPA. Arthritis Res Ther.

[27] Stoop J, Liu B, Shi J, Jansen D, Hegen M, Huizinga TWJ (2014). Antibodies specific for carbamylated proteins precede the onset of clinical symptoms in mice with collagen Induced Arthritis. PLoS One.

[28] Mydel P, Wang Z, Brisslert M, Hellvard A, Dahlberg L, Hazen SL (2010). Carbamylation-dependent activation of T Cells: a novel mechanism in the pathogenesis of autoimmune arthritis. J Immunol.

[29] Derganova O, Martinez-Gamboa L, Egerer K, Bang H, Fredenhagen G, Roggenbuck D (2014). Selected cyclic citrullinated peptides derived from the sequence of mutated and citrullinated vimentin (MCV) are targeted by different antibodies subclasses in patients with rheumatoid arthritis in Russian patients. Clin Exp Rheumatol.

[30] Mathsson L, Mullazehi M, Wick MC, Sjxberg O, Van Vollenhoven R, Klareskog L (2008). Antibodies against citrullinated vimentin in rheumatoid arthritis: higher sensitivity and extended prognostic value concerning future radiographic progression as compared with antibodies against cyclic citrullinated peptides. Arthritis Rheum.

[31] Yee A, Webb T, Seaman A, Infantino M, Meacci F, Manfredi M (2015). Anti-CarP antibodies as promising marker to measure joint damage and disease activity in patients with rheumatoid arthritis. Immunol Res.

[32] Avdeeva A, Aleksandrova E, Novikov A, Smirnov A, Cherkasova M, Nasonov E (2014). The relationship of antibodies to modified citrullinated vimentin and markers of bone and cartilage destruction in rheumatoid arthritis. Int J Rheumatol.

[33] Burr M, Viatte S, Bukhari M, Plant D, Symmons D, Thomson W (2012). Long-term stability of anti-cyclic citrullinated peptide antibody status in patients with early inflammatory polyarthritis. Arthritis Res Ther.

[34] Robertsa JM, Veresa PR, Cochranc AK, Warnekea C, Burlingd IR, Yokelsond RJ (2011). Isocyanic acid in the atmosphere and its possible link to smoke-related health effects. PNAS.

