# Supplementary material for: Carbamylated vimentin represents a relevant autoantigen in Latin American (Cuban) rheumatoid arthritis patients
Source: Rheumatol Int. 2016 Apr 2;36:781–91. doi: 10.1007/s00296-016-3472-9 (PMC4873524; doi:10.1007/s00296-016-3472-9)
Supplement: Supplementary file 1 — Supplementary material 1 (DOCX 505 kb) [file 296_2016_3472_MOESM1_ESM.docx]

*SUPPLEMENTARY MATERIALS*

S1. Demographic and clinical characteristics of RA patients.

| **Variable** | **RA**  **n=101** | **RA<1 year**  **n= 33** | **RA>1 year**  **n= 68** |
| --- | --- | --- | --- |
| F, n (%) | 82 (81.2) | 24 (72.7) | 58 (85.3) |
| Age in years, m (IQR) | 51(43-61) | 49(38-57) | 52(44-61) |
| Smoker, n (%) | 20 (19.8) | 8 (24.2) | 12 (17,6) |
| Disease duration in years, m (IQR) | 3(1-8) | 1(0.5-1) | 7(3-12) |
| DAS28, m (IQR) | 5.1 (3.7-6.0) | 5.2 (3.6-6.3) | 5.1(3.7-6.0) |
| Remission: DAS28≤2.6; n (%) | 7 (6.9) | 1 (3.0) | 6 (8.8) |
| Low: DAS28 >2.6 and ≤3.2, n (%) | 12 (11.9) | 5 (15.2) | 7 (10.3) |
| Moderate: DAS28>3.2 and ≤5.1, n (%) | 33 (32.7) | 10 (30.3) | 23 (33.8) |
| High: DAS28 >5.1, n (%) | 49 (48.5) | 17 (51.5) | 32 (47.1) |
| HAQ, m (IQR) | 0.75 (0.25-1.25) | 0.61 (0.30-0.80) | 0.87 (0.25-1.38) |
| ESR, m (IQR) | 31 (16-55) | 40 (18-60) | 29 (16-55) |
| CRP positivity, n (%) | 61 (60.4) | 22 (66.7) | 39 (57.4) |
| Treatments |  |  |  |
| Methotrexate, n (%) | 77 (76.2) | 24 (72.7) | 53 (77.9) |
| Azathioprine, n (%) | 6 (5.9) | 0 | 6 (8.8) |
| Chloroquine, n (%) | 16 (15.8) | 5 (15.2) | 11(16.2) |
| Sulfasalazine, n (%) | 12 (11.9) | 3(9.1) | 9 (13.2) |
| Prednisone, n (%) | 87 (86.1) | 27 (81.8) | 60 (88.2) |

RA: rheumatoid arthritis. DAS28: disease activity score. ESR: Erythrocyte sedimentation rate. CRP: C-reactive protein. HAQ: Health Assessment Questionnaire Index. m: Median. n: Number of patients. IQR: Interquartile range.**A**

Unmodified native vimentin peptide

**G**

**G**

**V**

**Y**

**A**

**T**

**R**

**S**

**S**

**A**

**V**

**R**

**N-**

**-OH**

**G**

**R**

**V**

**Y**

**A**

**T**

**XX**

**S**

**S**

**A**

**V**

**R**

**N-**

**-OH**

Mutated and modified vimentin peptide

**B**

| Sequence | Name | Code designation |
| --- | --- | --- |
| G**G**VYAT**X**SSAVR | native vimentin peptide | - |
| G**R**VYAT**R**SSAVR | mutated vimentin peptide | P62 |
| G**R**VYAT**X**SSAVR | Mutated,citrullinated peptide (X = citrulline) | P18 |
| G**R**VYAT**X**SSAVR | Mutated,carbamylated peptide (X = Homocitrulline) | HC52 |

S2. Synthesized vimentin peptides

A: Illustration of the backbone peptide sequence used for the ELISA. Peptides are identical in length, with differences between them that are indicated with labels.

B: Amino acid sequence of used vimentin peptides. According to the UniprotReferenzsequenz P08670-1 we synthesized vimentin peptide position aas 58-69 and incorporated the following modifications.


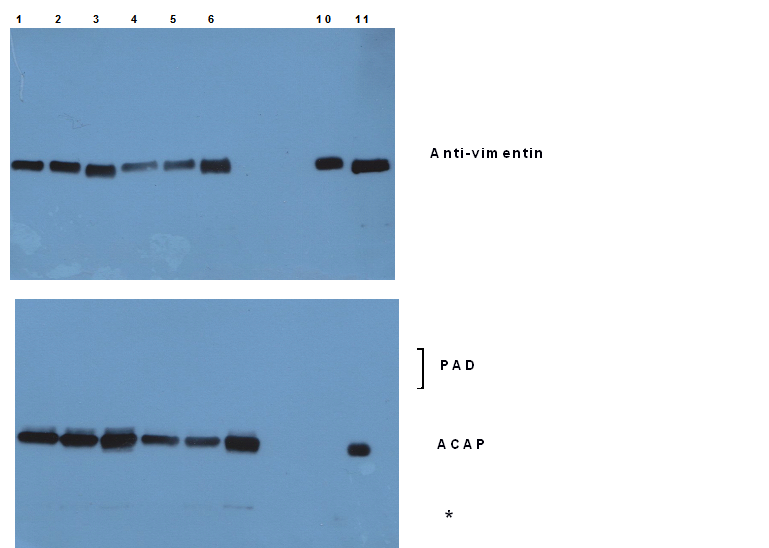


S3. Kinetic and specificity of citrullination of recombinant human vimentin with peptidyl arginine deiminase from rabbit skelatal muscle (Sigma, Germany) in vitro.

Lane **1- 6**, Recombinant human vimentin (2 mg/ml) was co-incubated with rabbit PAD (lane 1- 3 = 20 units PAD/ml; lane 4 - 6 = 40 units PAD/ml) in the presence of 10 mM CaCl_2_ at 37°C for 30 (**1**); 60 (**2**) and 120 (**3**). After termination of the reactions, the samples were electrophoresed and noncitrullinated and citrullinated proteins were detected by immunoblotting**(A)** with commercial anti–vimentin (H-84; Santa Cruz Biotechnology Inc., Heidelberg, Germany), and **(B)**Anti.citrullinated Protein antibodies (ACPA) sera pool from the previously published cohort (21).

**Note:** A thin low molecular weight protein band (asterix, *****) staining was detectable after 120 minutes with 40 units of PAD, but no autocitrullination of the PAD enzyme in the molecular weight range (~ 80 kDa)below vimentin is observed.

Lane 10 is the positive citrullinatedvimentin and lane 11 the un-modified negative control. To exclude any contamination two lanes were left between reference samples and the citrullination kinetic experiment.

1 2 3 1 2 31 2 3

A B C

S4. Analysis of untreated (lane 1), citrullinated (lane 2)and citrullinated plus carbamylated vimentin (lane 3) by SDS-PAGE electrophoresis with (A) Coomassie blue staining and immunoblotting with (B) anti-vimentin control antibody or (C) purified ACPA.


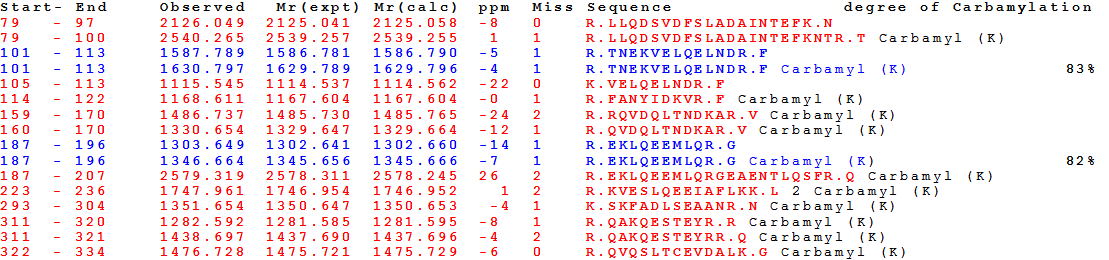


S5. Analysis of carbamylated vimentin

Recombinant human human vimentin was treated with 0.1 M cyanate to carbamylate the lysine amino acid residues. Resulting samples were digested with trypsin and the mass spectra of tryptic peptides were measured with a MALDI-TOF mass spectrometer (Ultraflex III TOF/TOF, BrukerDaltonics, Proteosys AG, Mainz, Germany). The m / z values of the peptides were compared with protein databases (NCBI, Mascot browser) and as a result, the tryptic peptides were determined to be assigned proteins (peptides mass fingerprint, PMF). In addition, MS/MS sequencing were (with the Ultraflex III TOF / TOF) performed on selected peptides.

Calculation of relative amounts of lysine and homocitrulline residues in each peptide was done by comparison to the quantitated synthetic peptide standard. Representative sequences and calculation of two peptides was shown: Peptide 101-113, Lys104= 83% and peptide 187-196, Lys189 = 82% carbamylation.


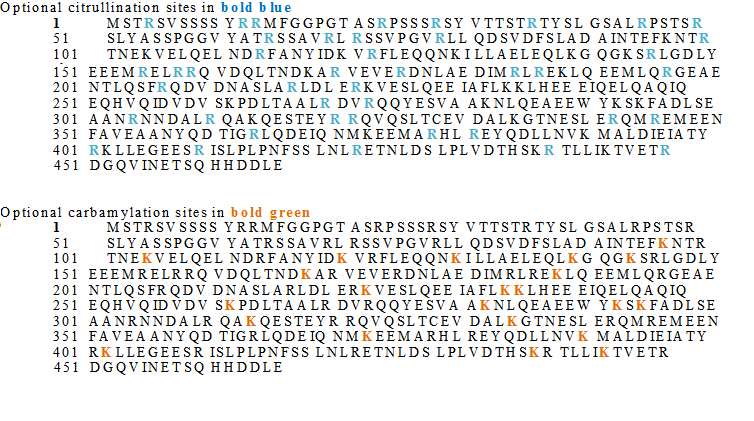
S6. Modification positions for citrullination (modified arginine R) and carbamylation (modified lysine K).

S7. Frequency of anti-MCV IgG, anti-carbVIMIgM and anti-CCP2 antibodies in disease and healthy controls.

| **Group** | **Anti-MCV IgG N (%)** | **Anti-carbVIMIgMN (%)** | **Anti-CCP2**  **N (%)** |
| --- | --- | --- | --- |
| Healthy controls (n=51) | 12 (23.5) | 2 (3.9) | 1 (2.0) |
| Undifferentiated arthritis (n=11) | 4 (36.4) | 1 (9.1) | 0 |
| SLE (n=10) | 3 (30.0) | 3 (30.0) | 1 (10.0) |
| MCTD (n=7) | 1 (14.3) | 2 (28.6) | 2 (28.5) |
| Psoriatic arthritis (n=3) | 0 | 1 (33.3) | 0 |
| Spondyloarthritis (n=2) | 0 | 0 | 0 |
| Autoimmune myositis (n=2) | 2 (100.0) | 0 | 1 (50.0) |
| Systemic sclerosis (n=3) | 0 | 0 | 0 |
| Rheumatic polymyalgia. (n=1) | 0 | 0 | 0 |
| Sjögren’s syndrome (n=1) | 0 | 0 | 0 |
| Viral hepatitis related arthralgia (n=10) | 3 (30.0) | 1 (10.0) | 0 |

MCV: mutated citrullinatedvimentin. carbVIM: carbamylatedvimentin. CCP2: cyclic citrullinated peptides of second generation. Ig: immunoglobulin. HC: Healthy controls.n: Number of positives patients. UA: Undifferentiated arthritis. Systemic lupus erythematosus. MCTD: mixed connective tissue disease. PA: Psoriatic arthritis. SP: Spondyloarthritis.AM: Autoimmune myositis. SS: Systemic sclerosis.RP: Rheumatic polymyalgia.SS: Sjögren’ssyndrome.SLE: VHRA: Viral hepatitis related arthralgia.

S8. Correlation coefficients between RF, ESR, and DAS 28 with autoantibodies against citrullinated and carbamylated sequences of vimentin and anti-CCP2 in RA patients.

|  | **Anti-CCP2** | **Anti-MCV IgG** | **Anti-carbVIMIgM** | **Anti-carbMCVIgM** |
| --- | --- | --- | --- | --- |
| Early RA (n=33) | | | | |
| RF | 0.7115* | 0.6804* | 0.3543* | 0.5053* |
| ESR | 0.2637* | 0.2591* | 0.2420* | 0.1997 |
| DAS 28 | 0.2332 | 0.2159 | 0.2130 | 0.2163 |
| Established RA (n=68) | | | | |
| RF | 0,6654* | 0,6515* | 0.3229* | 0,4200* |
| ESR | 0.1978 | 0.1282 | 0.0631 | 0.0668 |
| DAS 28 | 0.2778* | 0.1799 | 0.0881 | -0.0114 |

RA: rheumatoid arthritis. DAS 28: disease activity score. ESR: erythrocyte sedimentation rate. MCV: mutated citrullinatedvimentin. carbMCV: carbamylated mutated citrullinatedvimentin. carbVIM: carbamylatedvimentin. RF: rheumatoid factor. CCP2: cyclic citrullinated peptides of second generation. Ig: immunoglobulin. n: Number of patients.*p < 0.05
